# Influence of macrophage polarization on the effectiveness of surgical therapy of peri-implantitis

**DOI:** 10.1186/s40729-021-00391-2

**Published:** 2021-11-12

**Authors:** Maria Elisa Galarraga-Vinueza, Karina Obreja, Chantal Khoury, Amira Begic, Ausra Ramanauskaite, Anton Sculean, Frank Schwarz

**Affiliations:** 1 Department of Oral Surgery and Implantology, Johann Wolfgang Goethe-University, Carolinum, Theodor-Stern-Kai 7, 60596 Frankfurt am Main, Germany; 2 Post-Graduate Program in Implant Dentistry (PPGO), Federal University of Santa Catarina (UFSC), Florianópolis, SC Brazil; 3 Dentistry Faculty, Universidad de Las Américas (UDLA), Quito, Ecuador; 4 Department of Periodontology, School of Dental Medicine, University of Bern, Bern, Switzerland

**Keywords:** Biopsy, Dental implant, Immuno-histochemistry, Peri-implantitis, Macrophage polarization, Combined surgical therapy

## Abstract

**Purpose:**

To evaluate the influence of macrophage expression and polarization on the effectiveness of surgical therapy of peri-implantitis over a 6 month follow-up.

**Methods:**

A total of fourteen patients (*n* = 14 implants) diagnosed with peri-implantitis underwent access flap surgery, granulation tissue removal, implantoplasty, and augmentation at intra-bony components using a natural derived bone mineral and application of a native collagen membrane during a standardized surgical procedure. Granulation tissue biopsies were prepared for immunohistochemical characterization and macrophage polarization assessment. M1 and M2 phenotype expression was identified and quantified through immunohistochemical markers and histomorphometrical analyses. Clinical evaluation and data collection were performed initially and after a healing period of 6 months. Statistical analyses were performed to associate infiltrated area, macrophage, and M1/M2 phenotype influence on peri-implant tissue healing parameters after a 6-month follow-up.

**Results:**

Mean infiltrated compartment (ICT) values occupied a total percentage of 70.3% ± 13.0 in the analyzed granulation tissue biopsies. Macrophages occupied a mean area of 15.3% ± 7.0. M1 and M2 phenotypes were present in 7.1 ± 4.1% and 5.5 ± 3.7%, respectively. No statistically significant difference was observed between M1 and M2% expression (*p* = 0.16). The mean M1/ M2 ratio amounted to 1.5 ± 0.8. Surgical therapy was associated with statistically significant reductions in mean bleeding on probing (BOP), probing depth (PD) and suppuration (SUPP) scores at 6 months (*p* < 0.05). Linear regression analyses revealed a significant correlation between macrophage expression (CD68%) and changes in PD scores and M1 (%) expression and changes in mucosal recession (MR) scores at 6 months.

**Conclusions:**

The present data suggest that macrophages might influence peri-implant tissue healing mechanisms following surgical therapy of peri-implantitis over a short-term period. Particularly, changes in PD and MR scores were statistically significantly associated with macrophage expression and phenotype.

**Graphical Abstract:**

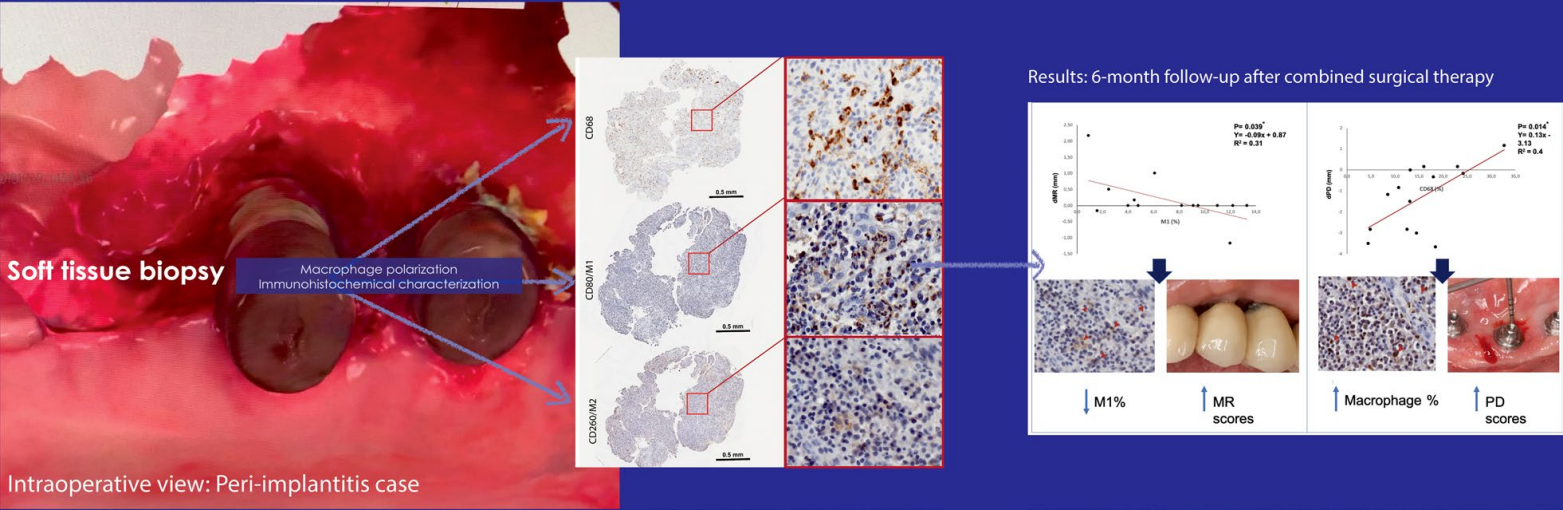

## Background

Emerging evidence indicates that macrophages may play a central role in the pathogenesis of peri-implant diseases [1–6]. It is well documented that macrophages can be triggered by certain molecules to polarize into different phenotypes and become protagonists of either disease progression or resolution [7–9]. M1 and M2 phenotypes have been deeply investigated and known to be pro-inflammatory or anti-inflammatory, respectively [1, 4, 6, 8]. A recent histological study revealed that M1 phenotype expression was significantly higher in peri-implantitis biopsies when compared with biopsies taken at periodontitis sites [2]. Moreover, a previous immunohistochemical investigation pointed out that M1 phenotype expression and subsequently the ratio of M1/M2 was significantly associated with the severity of peri-implantitis [5].

While these data suggest that macrophages could possibly play a critical role in the progression of peri-implantitis, it is currently unknown how a specific M1/M2 ratio may also affect wound healing following therapy. In fact, the available evidence indicates that nonsurgical therapy has a limited efficacy, which was particularly true for advanced peri-implantitis sites [10, 11].

While various surgical approaches (i.e. open flap debridement along with either resective and/ or reconstructive techniques) were proven to improve the clinical outcomes over nonsurgical treatment procedures, the reported efficacy varied considerably [12–14].

It might be hypothesized that these variations might be due to the specific pro-inflammatory environment generated by a high M1 phenotype expression.

Therefore, the present retrospective study aimed at evaluating the influence of macrophage expression and polarization on the effectiveness of a standardized combined surgical therapy of peri-implantitis.

## Materials and methods

### Study design

This retrospective study included a total of *n* = 14 patients who attended the Department of Oral Surgery and Implantology, Goethe University, Frankfurt, Germany (period of recruitment: May–November 2019), underwent a standardized combined surgical therapy of peri-implantitis, and were observed over a 6-month follow-up period.

The study protocol No. 92/19 was approved by the ethics committee of Goethe-University, Frankfurt-Germany, 2019 and considered the Helsinki Declaration of 1975, as revised in 2013. The following reporting considered the checklist items of the STROBE statement.

### Case definition

Peri-implantitis was defined as the combination of “bleeding on gentle probing (BOP) with/without suppuration (SUPP), probing depths (PD) ≥ 6 mm, and radiographic marginal bone loss (MBL) (i.e. “interproximal bone levels ≥ 3 mm apical of the most coronal portion of the intraosseous part of the implant”)” [15].

### Selection and enrollment of participants

#### Inclusion criteria

For patient selection, the following inclusion criteria were considered:

1. minimum age of 18 years old
2. partially/totally edentulous patients rehabilitated with implant-supported prosthesis that were diagnosed with peri-implantitis and underwent a combined surgical therapy.
3. presence of granulation tissue biopsies obtained during surgery
4. plaque index < 1 [16] before surgery
5. patients that were followed-up for 6 months and presented all clinical records

#### Exclusion criteria

The following exclusion criteria were considered:

1. incomplete clinical records over a 6-month follow-up period
2. untreated periodontal disease
3. pregnant or lactant women
4. autoimmune or/and inflammatory diseases
5. uncontrolled diabetes (HbA1c > 7)
6. corticosteroid therapy

### Clinical examination

The following clinical parameters were evaluated before and after a healing period of 6-months at each implant site using a periodontal probe (PCV12PT Hu-Friedy Inc., Chicago, IL, USA): (1) BOP evaluated as present if bleeding was evident within 30 s after probing, (2) PD as measured from the mucosal margin to the bottom of the probable pocket, (3) plaque index (PI) [16], (4) keratinized mucosa (KM) width, (5) mucosal recession (MR) as measured from the mucosal margin to the crown margin, and SUPP, evaluated as present if evident after probing and/or peri-implant palpation. All measurements were performed at six aspects per implant: mesio-vestibular, mid-vestibular, disto-vestibular, mesio-oral, mid-oral, and disto-oral by one calibrated examiner (K.O).

### Radiological examination

Radiological peri-implant bone loss at baseline was measured in a software program (Image J, Wisconsin, USA). MBL linear measurements were done following a previously described methodology [5] “by drawing a vertical line from the implant shoulder to the end of the defect at distal and mesial sites”. The measurement scale was set by the known implant length. Radiological assessment was done by one experienced examiner (M.E.G.).

### Surgical procedure and sample collection

**Fig. 1:**
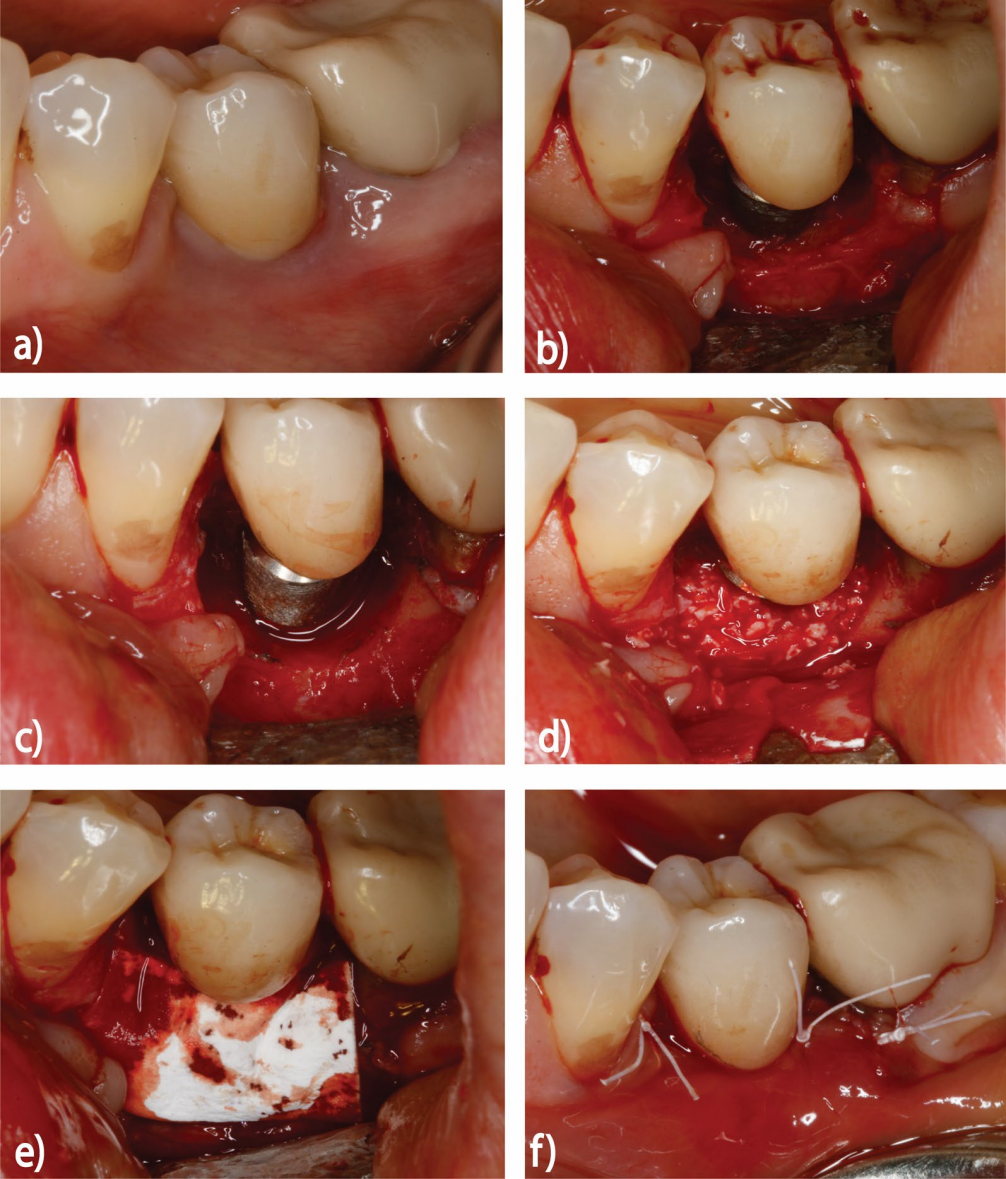
Schematic diagram showing the protocol for peri-implantitis combined surgical therapy: **a** initial situation before surgical therapy, **b** full thickness mucoperiosteal flaps raised at vestibular and oral aspects, **c** removal of granulation tissue and implant surface decontamination, **d**, **e** intrabony defect compartments were homogeneously filled using a natural bone mineral and covered with a native collagen membrane and **f** mucoperiosteal flaps were repositioned and adapted using non-resorbable double sutures

All patients received a pre-operative professional supra-gingival tooth/implant cleaning and treated according to an established and standardized protocol for combined surgical therapy of peri-implantitis [5, 17]. In particular, following local anesthesia (articaine, 1:200.000), buccal and lingual mucoperiostal flaps were raised to expose the defect area. Debridement and granulation tissue removal were accomplished using conventional plastic curettes (Straumann Dental Implant System; Institut Straumann AG, Basel, Switzerland). Soft tissue biopsies were collected and conserved in 4% buffered formalin for 24 h. Implantoplasty was accomplished at supracrestally and/ or bucally exposed implant surfaces using diamond burrs. The intrabony defect components were augmented using a natural bone mineral (BioOss spongiosa granules, particle size 0.25–1 mm; Geistlich, Wolhusen, Switzerland) (NBM) and covered by a native collagen membrane (BioGide; Geistlich) (Fig. 1).

### Sample process and immunohistochemistry

Granulation tissue biopsies taken from the defect compartment were analyzed according to an established methodology [5]. Biopsies were deposited in 70% ethanol at 4 ºC, dehydrated, and subsequently embedded in paraffin. Then, serial section (4 µm thick) were cut and mounted on glass poly-D-lysine coated slides. The paraffin-embedded sections were processed for immunohistochemical analyses. In particular, primary mouse monoclonal antibody against CD68 (1:200, NCL-L, Abcam, USA), rabbit monoclonal antibody against CD80 (1:200, EPR11572, Abcam, USA) and rabbit polyclonal mannose receptor antibody against CD206 (1:300, AB64693, Abcam, USA) were applied to detect macrophages, M1 and M2 phenotypes, respectively. Negative controls were implemented by replacing the primary antibody with non-immune serum.

### Histological examination

Each specimen was entirely scanned (40 × magnification) using a digital virtual light microscopy system (Nikon, NIS, Basic Research; Nikon E200 microscope, Japan) and evaluated according to an established methodology [5].

In brief, the surface area (µm^2^) of the infiltrated compartment (ICT) was delineated and the total stained (brown pigmented) surface area was divided by the ICT surface area to determine the positive cell proportions (%) of macrophages, M1 and M2 phenotypes per sample. To confirm that the stained areas for CD80 and CD206 markers corresponded to macrophage positive cells, every analyzed region was simultaneously observed for CD80 and CD68 or CD206 and CD68 markers. If the stained areas (cells) for M1 or M2 markers did not match with the stained areas for CD68 positive cells, they were not considered for the analysis [5].

### Statistical analysis

Statistical analysis was done using a commercially available software program (SPSS, 19.0, Chicago, IL, USA). Mean values, standard deviations, and confidence intervals were calculated at the patient level (equivalent to implant level). The Shapiro–Wilk test was used to assess data distribution. The paired t-test was applied to analyze clinical parameter changes at baseline and at 6 months and to determine the difference between M1 and M2 expression in the analyzed specimens. Linear regression analyses were executed to assess the relationship between baseline ICT (%), CD68%, M1%, M2%, as well as M1/M2 ratio and the changes in BOP, PD, MR, and KM scores at 6 months. The alpha error was set at 0.05.

## Results

This study included a total of 14 patients (10 females and 4 males) (*n* = 14 implants, mean age: 66.3 years; range: 53 to 78 years). Three (21.4%) patients reported a history of periodontal disease and four patients (28.5%) reported to smoke occasionally. The 14 evaluated implants were bone level and had an internal conical connection system. The mean implant function was 10.0 ± 6.2 years and the mean MBL at baseline amounted to 3.2 ± 1.3 mm. Implant site characteristics and frequencies are summarized in Table 1.

**Table 1:** Description of implant site characteristics and frequency distributions

Site characteristics	Number (*n* = 14)	Percentage (%)
Region		
Anterior	5	35.7
Posterior	9	64.3
Jaw		
Maxilla	8	57
Mandible	6	43
Bone grafted site		
Yes	5	35.7
No	9	64.3
Soft tissue grafted site		
Yes	1	7
No	13	93
Prosthesis retention type		
Screwed	3	21.4
Cemented	11	78.6
Prosthesis extension		
Single	9	64.3
Multiple	5	35.7

### Immunohistochemical assessment

**Fig. 2:**
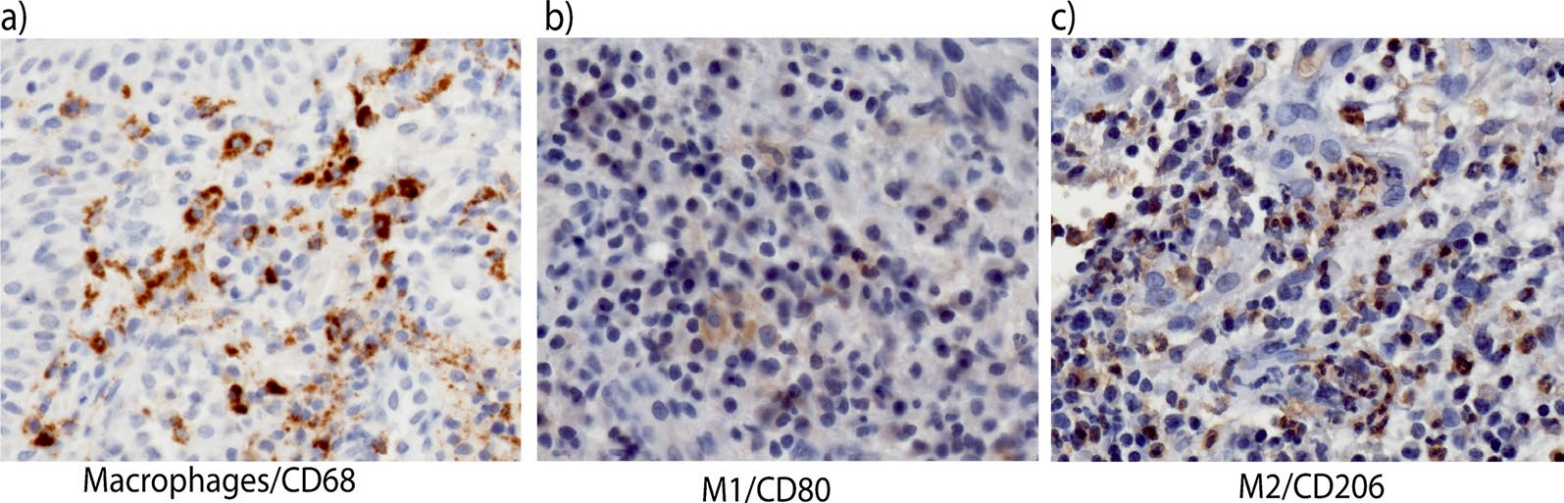
Histological sections depicting the antigen- reactivity for **a** macrophages (CD68), **b** M1 (CD80) and **c** M2 (CD206) phenotypes

Mean ICT values in the analyzed granulation tissue biopsies amounted to 70.3% ± 13.0. Macrophages identified with the CD68 marker occupied a mean proportioned area of 15.3% ± 7.0 (Fig. 2a). M1 and M2 phenotypes were present in 7.1 ± 4.1% and 5.5 ± 3.7%, respectively (Fig. 2b and c). No significant difference was observed between M1 and M2% expression (*p* = 0.16). The mean M1/ M2 ratio amounted to 1.5 ± 0.8.

### Clinical measurements

Postoperative wound healing was commonly uneventful and no implants were lost during follow-up.

Clinical parameters at baseline and after 6 months are summarized in Table 2.

**Table 2:** Clinical parameters (mean, SD, confidence interval and difference values) before surgical procedure and after a 6 month follow-up period (patient level) (*n* = 14). *p < 0.05

Clinical parameter	Baseline	95% CI	6 Months	95% CI	Changes
PI (mean)*	0.7 ± 0.46	0.45–0.97	0.2 ± 0.3	0.05–0.37	− 0.5 ± 0.47 *p* = 0.002
BOP (%)^†^	73.8 ± 30	58–89.5	17 ± 27	3–31.1	− 57 ± 40 *p* = 0.0001
PD (mm)^‡^	4.7 ± 1.58	3.86–5.5	3.4 ± 0.9	3–3.9	− 1.3 ± 1.5 *p* = 0.009
KM (mm)§	3.2 ± 2	2.19–4.3	3.3 ± 1.9	2.3–4.9	0.1 ± 1.6 *p* = 0.8
MR (mm)**	0.55 ± 1.2	− 0.08–1.2	0.73 ± 1.7	− 0.16–1.62	0.18 ± 0.7 *p* = 0.37
SUPP (%)^††^					
Yes	35		0		*p* = 0.012
No	65		100		

*Plaque Index

^†^Bleeding on Probing

^‡^Probing depth

^§^Keratinized mucosa

**Mucosal Recession

^††^Suppuration

All patients revealed low PI scores at baseline with further improvements of − 0.5 ± 0.47 at 6 months (*p* = 0.002). Surgical therapy was associated with marked and significant reductions in mean BOP (− 57.0 ± 40.0%; *p* = 0.0001) and PD (− 1.30 ± 1.50; *p* = 0.009) scores at 6 months. This was associated with a significant decrease in the frequency of SUPP positive implant sites from 35 to 0% (*p* = 0.012).

Minimal and non-significant changes were noted for mean MR and KM values, amounting to 0.18 ± 0.7 (*p* = 0.37) mm and 0.1 ± 1.6 mm (*p* = 0.8), respectively (Table 2).

### Regression analysis

**Fig. 3:**
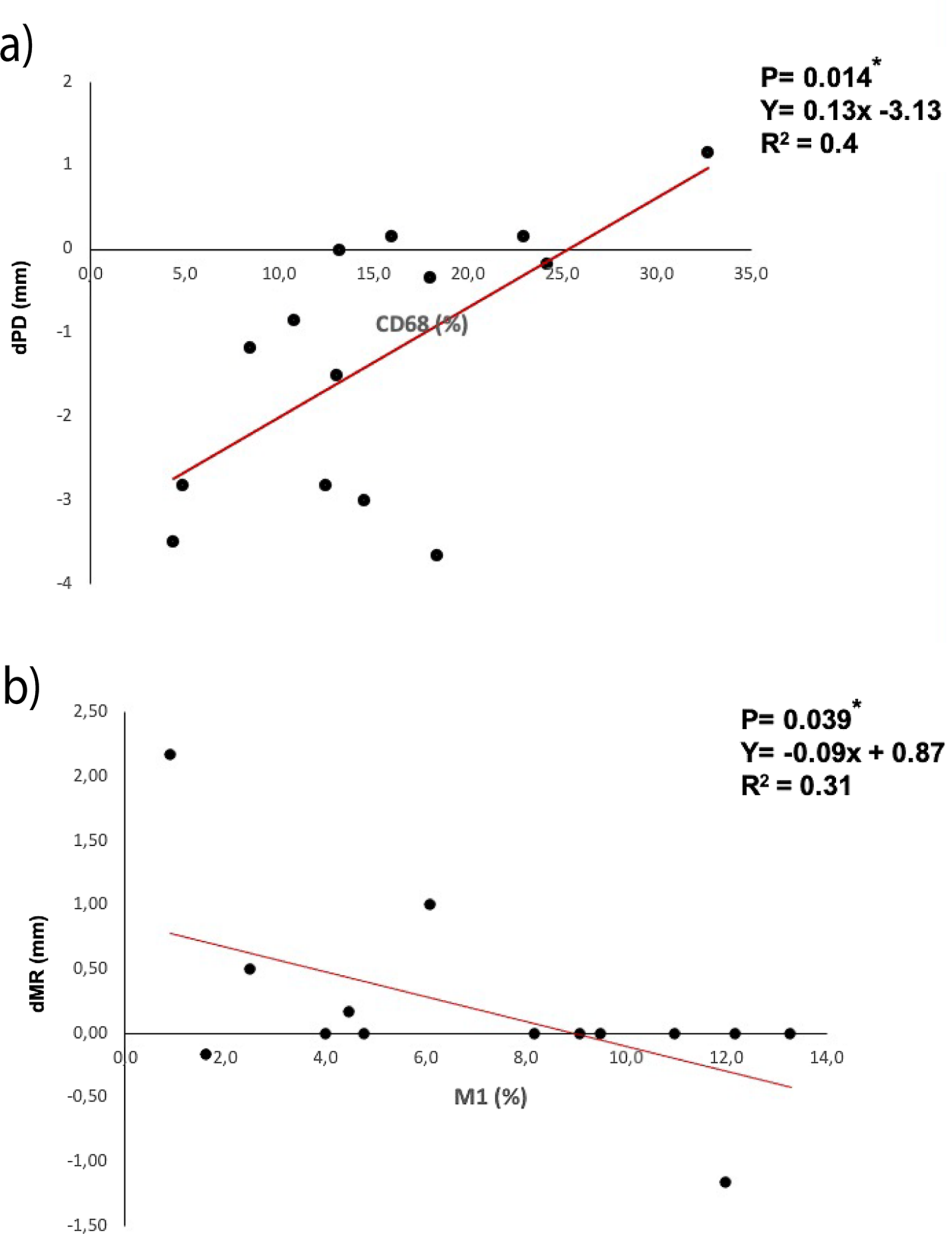
Linear regression plots representing a significant correlation between: **a** macrophage expression (CD68%) and changes in PD scores and **b** M1 (%) expression and changes in MR scores. *p < 0.05 considered for statistical significance

The linear regression analysis revealed a significant correlation between macrophage expression (CD68%) and changes in PD scores (*R*^2^ = 0.4; *p* = 0.014), indicating that lower CD68% scores at baseline were associated with higher reductions in PD scores at 6 months (Fig. 3a).

A statistically significant correlation was also noted between M1 (%) expression and MR scores (*R*^2^ = 0.31; *p* = 0.039), indicating that lower M1% scores at baseline were associated with higher increases in MR scores at 6 months (Fig. 3b).

## Discussion

The present retrospective study aimed at assessing the influence of macrophage expression and polarization on the effectiveness of surgical therapy of peri-implantitis after a 6 month follow-up period.

Basically, it was observed that the combined surgical procedure was associated with statistically significant reductions in mean BOP, PD and SUPP scores at 6 months. Furthermore, the analysis revealed that macrophage expression and changes in PD scores were statistically significantly correlated, indicating that lower CD68% scores at baseline were associated with higher reductions in PD scores. Moreover, a statistically significant correlation between M1 expression and MR scores revealed that lower M1% scores at baseline were associated with higher increases in MR scores.

To the author`s best knowledge, these are the first data on a potential correlation between macrophage polarization and changes in clinical outcomes following surgical therapy of peri-implantitis. In general, there is limited evidence on how macrophages residing in peri-implantitis lesions might affect or influence disease progression or resolution. A recent investigation revealed that macrophage polarization to M1 phenotype was statistically significantly associated with disease severity. In particular, advanced sites (i.e. MBL > 50% of implant length) had a statistically significantly higher M1% expression when compared with moderate or initial sites [5]. Accordingly, M1 phenotype which has been reported to be pro-inflammatory and induce osteolytic effects in several studies [3, 4, 18] could possibly be a significant factor in pathogenesis of peri-implantitis. Furthermore, another immunohistochemical analysis revealed a statistically significantly higher M1 expression in biopsies taken at peri-implantitis when compared with periodontitis sites. The latter study claimed that peri-implantitis lesions do progress faster than periodontitis lesions due to the quantity and phenotypic expression of macrophages [2]. Unfortunately, no further evidence regarding the potential role of macrophages in the pathogenesis or resolution of peri-implantitis lesions exists, thus limiting any comparisons of the presented results with previous literature.

However, basic research on wound healing mechanisms have indicated that macrophage’s plasticity and polarization are essential to achieve an effective wound repair and that essentially the ability of the M2 phenotype to trigger angiogenic responses and endothelial growth factor expression could accelerate the resolution of inflammatory lesions and subsequently tissue repair [19, 20]. While the present study, for obvious ethical reasons, could not consider changes in the M1 and M2% expression following therapy, it is assumed that the noted statistically significant improvements in all clinical parameters (i.e. BOP, PD, SUPP) investigated may also be linked with a resolution of the pro-inflammatory component of the associated lesions. In this context, it must be emphasized that these clinical improvements were comparable with those reported in previous clinical studies evaluating a similar combined surgical treatment procedure [21, 22]. However, the latter studies also indicated that a disease resolution could not be obtained in all patients investigated, as evidenced by the presence of e.g. high residual PD scores at 6 to 12 months following therapy.

This observation is also supported by the present analysis, suggesting that higher CD68% scores at baseline were associated with lower PD reductions at 6 months. One potential explanation for this finding may be the fact that macrophage and M1% expression was significantly associated with higher PD values [5], which in turn revealed lower changes following therapy.

When further analyzing the present data, there was also a significant association noted between M1% expression and changes in MR scores at 6 months. It is reasonable to assume that a higher M1 expression was associated with a more intense swelling of the peri-implant soft tissues at baseline, thus leading to a more pronounced tissue remodeling and subsequently increases in MR following therapy at respective sites. In fact, recent data have indicated that the peri-implant mucosa undergoes considerable volumetric changes after combined surgical therapy, with mean thickness changes (i.e. loss) amounting to − 0.11 and − 0.28 mm at 1 and 6 months. These changes were particularly pronounced at the marginal aspect of the peri-implant mucosa [14].

Nevertheless, the present study has certain limitations such as a highly variable population (i.e. smokers & patients with history of periodontal disease), missing control group due to ethical considerations, and a small sample size, therefore, the aforementioned associations showed possible tendencies between macrophage expression and changes in PD and MR scores. These tendencies should be further investigated in future studies.

Within its limitations, the present study suggest that macrophages might influence peri-implant tissue healing mechanisms following surgical therapy of peri-implantitis over a short-term period. Particularly, changes in PD and MR scores appear to possibly be linked to macrophage expression and phenotype.

## Data Availability

Not applicable.
